# Cardiovascular autonomic failure correlates with cutaneous autonomic denervation in PD and MSA

**DOI:** 10.1007/s10286-025-01154-4

**Published:** 2025-09-12

**Authors:** Shiwen Koay, Vincenzo Provitera, Ekawat Vichayanrat, Giuseppe Caporaso, Fernanda Valerio, Annamaria Stancanelli, Ilaria Borreca, Fiore Manganelli, Lucio Santoro, Maria Nolano, Valeria Iodice

**Affiliations:** 1 Department of Translational Neuroscience and Stroke, University College London Queen Square Institute of Neurology, Queen Square, London, WC1N 3BG UK; 2 Autonomic Unit, The National Hospital for Neurology and Neurosurgery, London, UK; 3 Neurology Department, Skin Biopsy Laboratory, Istituti Clinici Scientifici Maugeri IRCCS, Telese Terme, Italy; 4 Department of Neurosciences, Reproductive Sciences and Odontostomatology, University Federico II of Naples, Naples, Italy

**Keywords:** Orthostatic hypotension, Autonomic failure, Parkinson’s disease, Multiple system atrophy, Punch skin biopsy

## Abstract

**Purpose:**

Cardiovascular autonomic failure and neurogenic orthostatic hypotension (nOH) are common and disabling in Parkinson’s disease (PD) and multiple system atrophy (MSA). Recent studies have shown evidence of postganglionic autonomic denervation in MSA as well as PD. We aimed to characterise the relationship between nOH, autonomic failure and postganglionic denervation in PD and MSA. We hypothesised that postganglionic autonomic denervation contributes to the development of nOH and correlates with the severity of cardiovascular autonomic failure.

**Methods:**

We assessed 57 patients (37 PD, 20 MSA, median 64 [IQR 59–70] years) with cardiovascular autonomic testing; dynamic sweat testing; plasma noradrenaline levels; skin biopsies for quantification of intraepidermal, pilomotor and sudomotor innervation; and autonomic symptom questionnaires.

**Results:**

Overall, 78% of patients with MSA and 36% with PD had nOH ≥ 20/10 mmHg. The MSA group had more severe nOH, sudomotor dysfunction and cutaneous denervation, with higher supine noradrenaline than the PD group. Only supine noradrenaline differed between MSA and PD with nOH subgroups (*P* = 0.04). Overall, patients with nOH demonstrated more severe (1) cardiovascular autonomic failure, with reduced pressor responses to isometric exercise, deep breathing and Valsalva ratio; (2) intraepidermal, pilomotor and sudomotor denervation; and (3) autonomic symptoms and Hoehn–Yahr grade. The severity of nOH and cardiovascular autonomic failure correlated with autonomic denervation, patient symptoms and Hoehn–Yahr grade (*ρ* ≥ 0.50).

**Conclusions:**

nOH was associated with cutaneous autonomic denervation in both PD and MSA, with correlations between cardiovascular autonomic failure, cutaneous denervation and Hoehn–Yahr grade. Postganglionic autonomic denervation may contribute to nOH in PD and MSA, and affect responses to therapeutic agents.

**Supplementary Information:**

The online version contains supplementary material available at 10.1007/s10286-025-01154-4.

## Introduction

Cardiovascular autonomic failure and neurogenic orthostatic hypotension (nOH) are common and disabling features of the neurodegenerative diseases Parkinson’s disease (PD) and multiple system atrophy (MSA) [1]. Severe symptomatic autonomic failure and nOH are associated with shortened survival and worse disease progression in both PD and MSA [2, 3]. Previous studies have shown biomarkers reflecting postganglionic adrenergic autonomic innervation, such as supine plasma noradrenaline levels and cardiac meta-iodobenzylguanine (MIBG) scintigraphy studies tend to be relatively preserved in MSA compared with PD [4, 5], suggesting that autonomic failure in MSA occurs due to degeneration and dysfunction of central autonomic networks. However, our group and others have found evidence of postganglionic sudomotor dysfunction and cutaneous somatic and autonomic denervation in MSA, suggesting both central and peripheral degeneration may contribute to the pathophysiology of autonomic failure in MSA [6–9].

Coon et al. reported sudomotor evaluation in 232 patients with MSA, using the quantitative sudomotor axon reflex test (QSART), which assesses postganglionic sympathetic sudomotor axon integrity, and thermoregulatory sweat testing (TST), which assesses whole-body sweating. The site of the lesion was preganglionic in 47% and mixed (preganglionic and postganglionic) in 41%, with increasing frequency of postganglionic sudomotor abnormalities over time [9]. Our group previously compared sudomotor and intra-epidermal nerve fibre density (IENFD) at the distal leg, thigh, and fingertip in 29 patients with MSA and 29 age- and sex-matched healthy subjects. IENFD was reduced at all sites in MSA compared with controls in a non-length-dependent manner. Sudomotor nerve fibre density was lower in MSA compared with control subjects at the thigh and distal leg, with pan-neuronal marker protein gene product 9.5 (PGP) and cholinergic-specific marker vasoactive intestinal peptide (VIP). All patients with MSA, even those with less than a year disease duration, showed reduced sudomotor nerve fibre density in at least two of three examined sites, with about 60% loss compared with control values. Functional sudomotor evaluation using the silastic imprint test with pilocarpine iontophoresis in a subset of ten patients showed a reduced density of activated sweat glands in MSA compared with controls, with the loss correlating with sudomotor nerve fibre density at the distal leg [6].

We subsequently evaluated 57 patients with PD and 43 with MSA-P using a more comprehensive assessment of postganglionic sudomotor function, the dynamic sweat test (DST), which evaluates sweat gland density, sweat output per area and sweat output per gland after pilocarpine iontophoresis, and found that both patients with MSA-P and PD had impairments in all parameters compared with controls, with more abnormal findings in patients with MSA-P. Sweat gland morphology was more abnormal in patients with MSA-P, with a significantly lower average volume of sweat glands compared with PD, and in both patient groups compared with controls. IENFD, pilomotor and sudomotor nerve fibre densities were reduced in both groups compared with controls, with reduced nerve fibre densities in MSA-P compared with PD. Pilomotor and sudomotor nerve density correlated with sweating function [7].

Previous studies have described cardiovascular autonomic testing and sudomotor testing in PD and MSA with skin biopsies but have not comprehensively evaluated the relationship between quantitative measures of cardiovascular autonomic failure and postganglionic autonomic innervation on skin biopsy [7, 10, 11]. We hypothesised that postganglionic autonomic denervation may contribute to the development of nOH and correlate with severity of cardiovascular autonomic failure. We aimed to define the relationship between nOH, cardiovascular autonomic failure and markers of postganglionic autonomic denervation, including plasma catecholamines, DST and cutaneous autonomic innervation on punch skin biopsy in MSA and PD. A secondary aim was to assess whether these biomarkers could differentiate between MSA and PD, and MSA and PD with nOH.

## Methods

### Patient recruitment and study registration details

Patients with at least clinically probable PD and MSA according to established consensus criteria were recruited from patients referred to (1) Autonomic Unit, the National Hospital of Neurology and Neurosurgery, London; (2) Neurology Division ‘ICS Maugeri’ IRCCS of Telese Terme; and (3) Neurology Department, University of Naples Federico II, Naples, between November 2017 and December 2019, as part of a multi-centre natural history and biomarker study in Parkinsonism. Diagnoses of PD and MSA were made by consultant neurologists responsible for the care of these patients using the 2015 MDS Clinical Diagnostic Criteria for Parkinson’s Disease [12] and the 2008 consensus statement on the diagnosis of multiple system atrophy [13]. Following the publication of the 2022 MDS Criteria for the Diagnosis of MSA [14], we have reviewed all cases to confirm they also met the latest diagnostic criteria for at least clinically probable MSA. All patients were followed up for at least 2 years. Any patients with an alternative diagnosis other than PD or MSA, or an unclear diagnosis, were excluded from the final analysis. Patients with conditions associated with peripheral neuropathies, including diabetes, HIV, connective tissue disorders and other toxic or metabolic disorders were also excluded.

The study protocol complied with the Declaration of Helsinki and was approved by the local institutional review boards (Fondazione G. Pascale no. ‘5/15 Maugeri’ and London Bridge Research Ethics Committee, REC reference 16/LO/1656). All subjects gave written informed consent to participate in the study. Study data were collected and managed using REDCap research electronic data capture tools hosted at University College London [15, 16].

### Clinical assessment and patient-reported outcomes

All patients were assessed by neurologists with movement disorders expertise, with recording of their motor examination and functional impairment using the Hoehn-Yahr scale and the Unified Multiple System Atrophy Rating Scale (UMSARS)/Unified Parkinson’s Disease Rating Scale (UPDRS) [17]. Sensory and autonomic symptoms were collected using the COMPASS-31 and SFN-SIQ [18, 19]. Disease duration was defined as time from onset of motor symptoms to the time of recruitment to the study.

### Imaging

Brain magnetic resonance imaging (MRI), DaTscans and cardiac MIBG were performed as part of the clinical work up as appropriate.

### Cardiovascular autonomic testing

All patients had a core protocol of cardiovascular autonomic testing performed with recording of blood pressure and heart rate:

1. At rest in the supine position
2. After 1-, 3- and 5-min standing or tilt.
3. Before and after isometric exercise (sustained handgrip for 3 min at a third of maximum voluntary contraction pressure).
4. With deep breathing, at a rate of 6 breaths/min.
5. With the Valsalva manoeuvre (forced expiration at 40 mmHg for 10 s). Beat-to-beat recordings of blood pressure and heart rate with analysis of the blood pressure profile in response to the Valsalva manoeuvre. Blood pressure recovery time (PRT), a marker of adrenergic control of total peripheral resistance, was defined as the time taken for the systolic blood pressure to recover from phase III of the Valsalva manoeuvre back to baseline [20]. The Valsalva ratio was calculated by dividing the maximal heart rate developed in response to blood pressure reduction induced by the Valsalva manoeuvre by the minimum heart rate occurring within 30 s of the peak heart rate [20].

All medications potentially affecting autonomic testing were stopped at least five half-lives prior to testing, and patients were instructed to consume only water for 4 h prior to testing. nOH was defined as a sustained fall of at least 20 mmHg systolic or 10 mmHg diastolic blood pressure within 3 min of standing or tilt according to consensus criteria [21], with an abnormal blood pressure profile in response to the Valsalva manoeuvre, or blunted HR increase during hypotension with ΔHR/ΔSBP ratio lower than 0.5 bpm/mmHg [22, 23]. Blood was collected via intravenous catheter in the supine and tilted position for measurement of plasma catecholamines using high performance liquid chromatography [24].

### Sudomotor testing

All patients underwent DST at the distal leg bilaterally. The DST uses pharmacological stimulation with pilocarpine to directly stimulate the M3 muscarinic receptors on cutaneous sweat glands, providing an estimation of postganglionic sudomotor function [25]. After iontophoresis with 1% pilocarpine solution, skin was coated with iodine and the formation of sweat gland imprints on starch covered tape was recorded. Density of activated sweat glands/cm^2^, sweat nL/min/cm^2^ and average sweat output/gland were calculated.

### Morphological analysis

All patients had 3-mm skin biopsies collected from the distal leg bilaterally and processed for indirect immunofluorescence according to standard procedures using a large panel of antibodies, including primary antibodies against collagen type IV (ColIV); protein gene product (PGP) 9.5, a pan-neuronal marker; dopamine-β-hydroxylase (DβH), a marker for noradrenergic fibres; vasoactive intestinal peptide (VIP), a marker for cholinergic fibres; and species-specific secondary antibodies coupled with Cy2 and Cy3 fluorophores [26]. Digital confocal images were acquired using a non-laser confocal system (Apotome2 Zeiss, Jena, Germany, EU).

Analysis of skin biopsy samples was performed by individuals blinded to the diagnosis of the patients. Intraepidermal nerve fibre (IENF) density was measured according to current guidelines [27]. Quantification of pilomotor and sudomotor nerve fibres using pan-neuronal and selective cholinergic and noradrenergic markers was performed as previously described [6, 28].

For the functional and morphological studies above, the average of both sides was calculated and taken as representative for each patient. Normative data was extracted from a database of 100 healthy volunteers.

### Statistical analysis

Statistical analysis was performed using R, version 3.6.0. Normality was assessed Shapiro–Wilk tests. Summary data has been presented as median (IQR) as some data was not normally distributed. Patient groups were compared with unpaired two-tailed *t*-tests/Mann–Whitney tests, and subgroup comparisons were preformed using ANOVA/Kruskal–Wallis tests with Tukey/Dunn post hoc tests with Bonferroni corrections for multiple comparisons as appropriate. Chi-squared tests were used to compare categorical data. Correlations were performed using Spearman tests. *P* < 0.05 was considered significant.

## Results

### Demographic and clinical data

Postganglionic sudomotor function, cutaneous autonomic innervation and distribution of neural phosphorylated synuclein of the patients recruited to this multicentre cohort study have been described in previous reports [7, 29]. This study focused on the cardiovascular autonomic testing and the relationship between quantitative autonomic biomarkers and markers of postganglionic autonomic denervation in patients with MSA and PD, with and without nOH.

In total, 57 patients were included in the final analysis: 37 with PD and 20 with MSA (13 with parkinsonian subtype and 7 with cerebellar subtype). Overall, 32% (18/57) were female and the median age was 64 years (IQR 59–70 years) at recruitment. The MSA and PD groups had similar sex distribution, age and disease duration at recruitment, but patients with MSA had greater disease severity as measured by the Hoehn-Yahr scale (4 [3–4] versus 1.5 [1–2], *P* < 0.001) (Table 1).

**Table 1:** Clinical details and cardiovascular testing in MSA and PD

	Median (IQR)		*P*-value
	MSA (*n* = 20)	PD (*n* = 37)	
Clinical details			
Age, y	63 (55–68)	65 (58–70)	0.48
Sex (F/M)	6/14	12/25	> 0.99
Disease duration, mo	30 (17–42)	21 (12–23)	0.10
UMSARS/UPDRS score	42 (36–54)	42 (33–50)	0.25
Hoehn–Yahr	4 (3–4)	1.5 (1–2)	< 0.001
COMPASS-31	32 (16–45)	18 (11–29)	0.12
SFN-SIQ	9 (8–10)	6 (4–10)	0.26
Cardiovascular testing			
Supine			
SBP, mmHg	134 (121–155)	134 (123–140)	0.31
DBP, mmHg	82 (76–86)	84 (79–90)	0.60
HR, bpm	62 (59–72)	66 (60–69)	0.71
SBP on standing			
1 min, mmHg	107 (97–127)	125 (113–131)	0.25
3 min, mmHg	104 (97–119)	127 (114–141)	0.03
5 min, mmHg	100 (90–108)	129 (111–137)	0.001
ΔSBP on standing			
1 min, mmHg	32 (14–41)	7 (1–20)	.03
3 min, mmHg	32 (19–65)	4 (− 2 to 19)	0.004
5 min, mmHg	35 (28–77)	10 (1–26)	0.004
Isometric exercise			
ΔSBP, mmHg	6 (−1 to 14)	25 (20–32)	< 0.001
ΔHR, bpm	5 (2–8)	9 (5–14)	0.04
HR_DB_, bpm	8 (6–11)	12 (8–18)	0.06
Valsalva ratio	1.20 (1.12–1.3)	1.19 (1.08–1.39)	0.89
Pressure recovery time, s	14.5 (7.4–23.6)	7.6 (2.8–14.6)	0.30
Plasma noradrenaline, pg/mL			
Supine	256 (240–303)	216 (158–223)	0.02
Tilted	293 (247–316)	261 (229–283)	0.50
Δnoradrenaline on tilt	11 (5–12)	67 (38–71)	0.05

*SBP* systolic blood pressure, *HR* heart rate

### Brain imaging

Brain MRI was performed as part of the clinical work up in 25 patients (9 with PD, 9 with MSA-P and 7 with MSA-C. Amongst the nine patients with PD, two patients had evidence of small vessel disease and seven patients had normal brain MRI. Amongst the nine patients with MSA-P, two patients had atrophy of the putamen, pons, middle cerebellar peduncle and cerebellum, or the hot-cross bun sign; three patients had more generalised cerebral atrophy; two patients had evidence of small vessel disease; and two patients had normal brain MRI. Amongst the seven patients with MSA-C, five patients had atrophy of the putamen, pons or middle cerebellar peduncle or the hot-cross bun sign; and two patients had more generalised volume loss within the cerebellum and posterior fossa.

DaTscan was performed in 20 patients (12 with PD and 8 with MSA). There was reduced availability of presynaptic dopaminergic transporter activity in keeping with dopaminergic system degeneration in all 12 patients with PD and 3 patients with MSA-P. Five patients with MSA-C did not have any specific evidence of nigrostriatal dopaminergic denervation.

### Cardiovascular autonomic testing

Compared with patients with PD, patients with MSA had a significantly greater fall in systolic blood pressure on standing (35 [28–77] versus 10 [1–26] mmHg by 5 min, *P* = 0.004), and reduced blood pressure and heart rate responses to isometric exercise (6 [−1 to 14] versus 25 [20–32] mmHg, *P* < 0.001; 5 [2–8] versus 9 [4–14] bpm, *P* = 0.04).

Of the 57 patients studied, 43 patients completed at least 3 min of standing or tilt challenge, allowing us to assess for sustained nOH according to consensus criteria [21]. The other 14 patients had assessment of blood pressure and heart rate supine and after 1 min standing only as part of routine clinical testing and were not included in the analyses comparing patients with and without nOH.

Overall, 23/43 (54%) patients fulfilled criteria for nOH: 78% (14/18) patients with MSA and 36% (9/25) with PD. Of these 23 patients, 15 patients had assessment of their BP profile with the Valsalva manoeuvre, and all 15/15 (100%) had reduced or absent phase II late blood pressure recovery, phase IV overshoot and prolonged PRT, consistent with impaired adrenergic function and nOH. The eight patients with nOH on standing who did not perform Valsalva manoeuvre had minimal HR rise with 3 min of standing, in keeping with nOH (median ΔHR 1 [ IQR −2 to 4] bpm; median ΔHR/ΔSBP −0.04 (IQR −0.05 to 0.21, ΔHR/ΔSBP < 0.5 indicates nOH) [22, 23].

Overall, regardless of the diagnosis, patients with and without nOH were similar in age, but patients with nOH had a longer disease duration (33 [20–44] versus 21 [11–23] months, *P* = 0.005). They reported more severe autonomic symptoms (COMPASS-31 score 33 [25–48] versus 17 [9–30], *P* = 0.006) and had a higher Hoehn–Yahr grade (3.5 [2.75–4] versus 1 [1–2],* P* = 0.001) (Supplementary Table 1). Compared with patients without nOH, patients with nOH had a more prolonged PRT (14.5 [8.9–22.4] s versus 2.5 [2.5–2.8] s, *P* = 0.02), attenuated pressor responses to isometric exercise (5 [−2 to 15] mmHg versus 22 [14–26] mmHg, *P* < 0.001; 4 [1–6] versus 11 [7–14] bpm, *P* = 0.004), HR_DB_ (7 [4–11] bpm versus 12 [8–18] bpm, *P* = 0.048) and lower Valsalva ratio (1.10 [1.03–1.18] versus 1.33 [1.21–1.42], *P* = 0.02), reflecting more widespread sympathetic and parasympathetic autonomic failure. Cardiovascular autonomic biomarkers did not differ significantly between the MSA with nOH and PD with nOH subgroups (Table 2).

**Table 2:** Subgroup comparisons

Variable	Median (IQR)				*P*-value	
	MSA + OH (*n* = 14)	MSA, no OH (*n* = 4)	PD + OH (*n* = 9)	PD, no OH (*n* = 16)	ANOVA	MSA + OH versus PD + OH
Clinical details						
Age, y	64 (55–68)	62 (59–66)	65 (58–74)	64 (60–69)	0.91	0.81
Sex (F/M)	3/13	1/3	2/7	8/8	0.35	
Disease duration, mo	39 (29–59)	16 (10–20)	23 (19–35)	21 (11–23)	0.009	> 0.99
Hoehn–Yahr	4 (3.5–4)	3 (2–3)	3 (2–3)	1 (1–2)	< 0.001	0.45
SFN-SIQ	10 (8–10)	9 (9–9)	13 (6–21)	7 (5–11)	0.54	> 0.99
COMPASS-31	33 (22–48)	22 (17–26)	36 (28–48)	18 (9–31)	0.07	> 0.99
Cardiovascular testing						
Supine						
SBP, mmHg	136 (127–166)	131 (120–139)	136 (134–146)	133 (123–139)	0.16	> 0.99
DBP, mmHg	82 (75–84)	82 (76–90)	89 (84–93)	82 (75–87)	0.17	0.47
HR, bpm	63 (57–73)	62 (60–63)	67 (63–70)	66 (63–70)	0.42	> 0.99
SBP on standing						
1 min, mmHg	104 (91–113)	122 (115–133)	118 (110–134)	122 (117–141)	0.21	> 0.99
3 min, mmHg	103 (86–106)	124 (117–132)	115 (104–136)	131 (118–142)	0.01	> 0.99
5 min, mmHg	95 (82–107)	116 (110–131)	109 (106–134)	131 (118–137)	< 0.001	0.18
ΔSBP on standing						
1 min, mmHg	38 (28–51)	−4^a^ (−9 to 8)	21 (6–59)	3 (−3^a^ to 16)	0.001	0.87
3 min, mmHg	37 (30–75)	5 (−2 to 10)	21 (19–58)	4 (−2^a^ to 7)	< 0.001	0.95
5 min, mmHg	40 (30–84)	−2^a^ (−4 to 8)	29 (23–44)	5 (1–11)	< 0.001	0.59
Isometric exercise						
ΔSBP, mmHg	5 (−2.5^a^ to 10.2)	11 (5–16)	10 (3–21)	25 (22–27)	< 0.001	0.56
ΔHR, bpm	7 (3–6)	7 (3–12)	1 (0–4)	12 (8–14)	0.001	> 0.99
HR_DB_, bpm	7 (4–9)	13 (11–16)	12 (4–17)	11 (8–18)	0.09	0.75
Valsalva ratio	1.20 (1.13–1.28)	1.33 (1.29–1.34)	1.14 (1.07–1.19)	1.37 (1.21–1.50)	0.09	> 0.99
PRT,^b^ s	14.5 (7.4–23.6)	–	17.2 (14.6–19.7)	2.7 (3.5–2.8)	0.08	> 0.99
Plasma NA, pg/mL						
Supine	256 (240–303)	–	158 (154–190)	232 (224–239)	0.04	0.04
Tilted	293 (247–316)	–	229 (174–211)	325 (304–345)	0.07	0.16
ΔNA on tilt	11 (5–12)	–	38 (22–55)	93 (80–106)	0.08	0.81
DST						
Glands/cm^3^	24 (19–40)	39 (25–46)	53 (41–55)	46 (34–57)	0.04	0.05
Output/gland, nL	3.1 (1.5–4.3)	4.6 (3.2–5.1)	3.0 (2.5–4.1)	2.7 (2.2–4.7)	0.85	0.95
Sweat output, nL/cm^3^	73 (26–145)	79 (22–166)	149 (119–194)	119 (38–190)	0.25	0.31
Skin biopsies						
IENFD, f/mm	1.8 (0.9–4.5)	5.0 (4.9–10.3)	5.9 (2.6–9.1)	7.8 (6.1–9.4)	0.005	0.23
Pilomotor nerve fibre density, f/mm						
PGP	28.8 (20.0–46.7)	52.6 (50.2–59.0)	45.6 (38.2–50.7)	53.7 (39.5–59.0)	0.02	0.77
VIP	0.0 (0.0–5.2)	25.2 (21.6–25.8)	6.6 (4.3–13.0)	9.8 (5.7–15.2)	0.004	0.30
DβH	4.5 (0–13.6)	42.0 (38.3–43.9)	21.6 (11.9–40.2)	33.6 (20.4–45.2)	0.01	0.35
Sudomotor nerve fibre density, nm/µm^3^						
PGP	0.7 (0.5–2.0)	1.9 (1.6–2.7)	3.1 (2.1–3.7)	2.8 (2.5–3.9)	0.02	0.13
VIP	0.6 (0.0–0.9)	1.3 (1.2–1.4)	1.3 (0.7–1.6)	1.6 (1.4–1.8)	0.006	0.54

*SBP* systolic blood pressure, *HR* heart rate, *PRT* blood pressure recovery time, *NA* noradrenaline, *PNFD* pilomotor nerve fibre density, *SNFD* sudomotor nerve fibre density

### Plasma noradrenaline

**Fig. 1:**
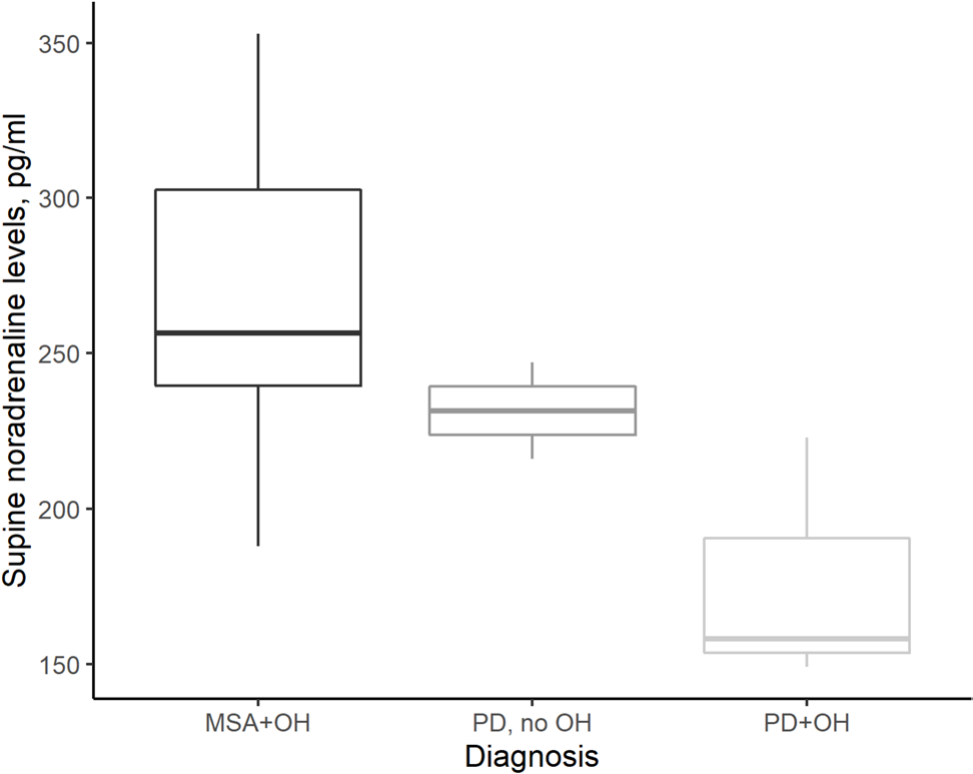
Supine noradrenaline levels in patients with MSA and PD, with and without OH. Supine noradrenaline levels were significantly lower in the PD + OH group compared with the MSA group (*P* = 0.04), with intermediate results in the PD without OH group. All patients with MSA who had plasma noradrenaline measured had OH

Seventeen patients had their plasma noradrenaline levels measured: 12 with MSA, all of whom had nOH, and 5 patients with PD (3 with nOH and 2 without nOH). Patients with MSA had normal supine noradrenaline (256 [240–303] pg/mL), with minimal rise on tilt (11 [5–12] pg/mL). In comparison, patients with PD with nOH had significantly lower supine noradrenaline (158 [154–190] pg/mL, *P* = 0.04), with modest rise on tilt (38 [22–55] pg/mL). Patients with PD without nOH had normal supine noradrenaline (232 [224–239] pg/mL) and preserved rise on tilt (93 [80–106] pg/mL) (Fig. 1).

### Postganglionic sudomotor function

Patients with MSA and PD had significantly lower sweat production/cm^2^, sweat output/gland and sweat gland density at the distal leg compared with healthy controls, with lower sweat gland density in MSA (28 [18–45] glands/cm^3^) compared with PD (46 [37–55] glands/cm^3^, *P* = 0.007; Table 3). There was no difference in postganglionic sudomotor function between patients with and without nOH (Table 4).

**Table 3:** Postganglionic sudomotor function and cutaneous innervation in MSA and PD

	Median (IQR)			*P*-value			
	MSA (*n* = 20)	PD (*n* = 37)	CTRL (*n* = 100) †	ANOVA	CTRL versus MSA	CTRL versus PD	MSA versus PD
Clinical details							
Age, y	63 (55–68)	65 (58–70)	61 (54–67)	0.22			
Sex (F/M)	6/14	12/25	49/51	0.10			
DST							
Glands/cm^3^	28 (18–45)	46 (37–55)	66 (62–85)	< 0.001	< 0.001	< 0.001	0.007
Output/gland, nL	3.1 (1.4–4.3)	3.5 (2.5–4.7)	11.4 (7.7–13.2)	< 0.001	< 0.001	< 0.001	0.09
Sweat output, nL/cm^3^	73 (22–139)	153 (102–261)	591 (363–871)	< 0.001	< 0.001	< 0.001	0.06
Skin biopsies							
IENFD, f/mm	3.3 (1.4–5.0)	7.8 (5.3–8.7)	12.1 (10.5–14.0)	< 0.001	< 0.001	< 0.001	0.22
Pilomotor nerve density, f/mm							
PGP	37.8 (22.0–50.6)	50.7 (38.2–65.4)	70 (66–90)	< 0.001	< 0.001	< 0.001	0.21
VIP	0.8 (0.0–17.9)	8.1 (2.6–21.5)	60.3 (52.4–69.4)	< 0.001	< 0.001	< 0.001	0.59
DβH	10.6 (0.4–30.3)	33.6 (20.4–44.1)	52 (49.5- 55)	< 0.001	< 0.001	< 0.001	0.18
Sudomotor nerve density, nm/µm^3^							
PGP	1.2 (0.6–3.2)	2.9 (2.3–3.7)	3.6 (3.1–3.9)	< 0.001	< 0.001	0.13	0.03
VIP	0.8 (0.3–1.2)	1.6 (1.1–1.9)	2.1 (1.9–2.7)	< 0.001	< 0.001	< 0.001	0.02

^†^DST data available for 50 healthy controls

**Table 4:** Postganglionic sudomotor function and cutaneous innervation in patients with and without OH

	Median (IQR)			*P*-value			
Variable	OH (*n* = 23)	No OH (*n* = 20)	CTRL (*n* = 100)	ANOVA	CTRL versus OH	CTRL versus no OH	No OH versus OH
DST							
Glands/cm^3^	37 (21–50)	45 (30–52)	66 (62–85)	< 0.001	< 0.001	< 0.001	0.59
Output/gland, nL	3.0 (2.3–4.3)	3.0 (2.2–4.9)	11.4 (7.7–13.2)	< 0.001	< 0.001	< 0.001	0.93
Sweat output, nL/cm^3^	113 (62–166)	119 (28–190)	591 (363–871)	< 0.001	< 0.001	< 0.001	1
Skin biopsies							
IENFD, f/mm	2.7 (1.3–5.4)	7.4 (5.1–10.0)	12.1 (10.5–14.0)	< 0.001	< 0.001	< 0.001	0.25
Pilomotor nerve density, f/mm							
PGP	36.6 (23.6–48.4)	53.1 (44.2–61.1)	70 (66–90)	< 0.001	< 0.001	0.01	0.09
VIP	2.8 (0–7.5)	11.8 (6.5–23.4)	60.3 (52.4–69.4)	< 0.001	< 0.001	0.002	0.13
DβH	10.6 (0.4–21.4)	36.1 (28.4–45.7)	52 (49.5–55)	< 0.001	< 0.001	0.06	0.08
Sudomotor nerve density, nm/µm^3^							
PGP	1.9 (0.7–3.6)	2.8 (2.2–3.7)	3.6 (3.1–3.9)	0.001	0.001	0.20	0.46
VIP	0.9 (0.1–1.3)	1.6 (1.4–1.8)	2.1 (1.9–2.7)	< 0.001	< 0.001	0.01	0.002

^†^DST data available for 15 healthy controls

### Cutaneous somatic and autonomic innervation

**Fig. 2:**
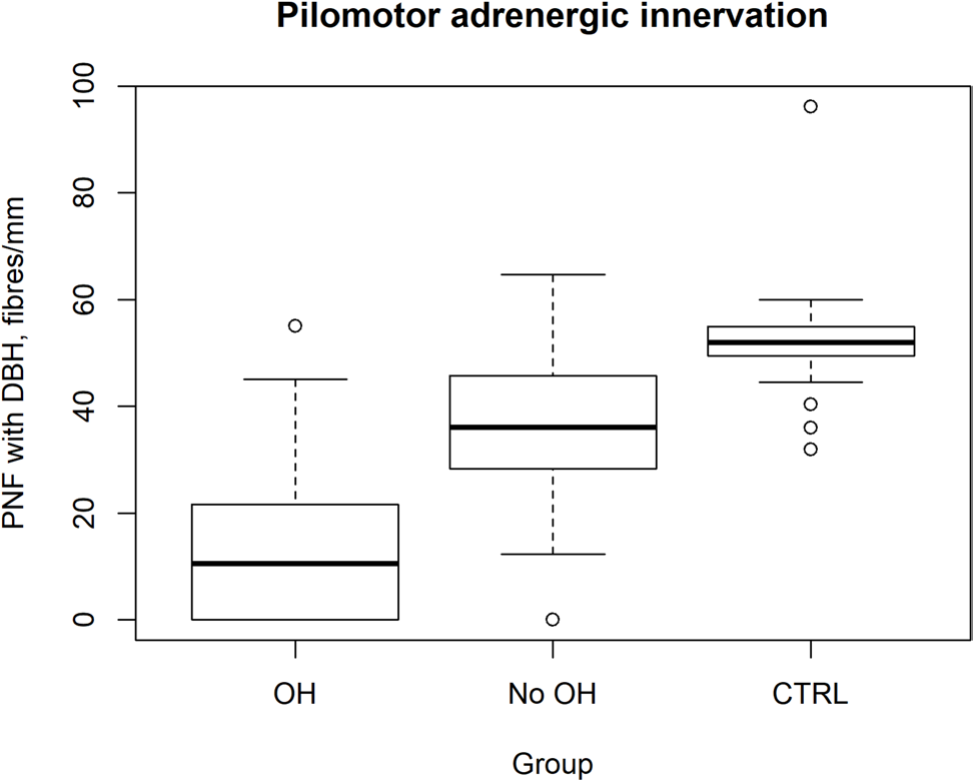
Comparison of pilomotor adrenergic innervation between patients with and without orthostatic hypotension and controls. Pilomotor adrenergic innervation was lowest in the OH group (10.6 [0.4–21.4] fibres/mm), followed by the non-OH group (36.1 [28.4–45.7] fibres/mm) and the control group (52 [49.5–55] fibres/mm), with a significant difference between the OH and control groups (*P* ≤ 0.001)

IENFD and pilomotor innervation using pan-neuronal marker PGP, cholinergic marker VIP and adrenergic marker DβH at the distal leg was reduced in MSA compared with controls (*P* < 0.001; Table 3). Sudomotor innervation with PGP was reduced in MSA (1.2 [0.6–3.2] nm/µm^3^) compared with PD (2.9 [2.3–3.7] nm/µm^3^, *P* = 0.03) and controls (3.6 [3.1–3.9] nm/µm^3^, *P* < 0.001). Sudomotor innervation with VIP was reduced in MSA (0.8 [ 0.3–1.2] nm/µm^3^) and PD (1.6 [1.1–1.9] nm/µm^3^) compared with controls (2.1 [1.9–2.7] nm/µm^3^, *P* < 0.001) and lower in MSA compared with PD (*P* = 0.02) (Fig. 2).

94% with MSA and 76% with PD had abnormal IENFD below 95% cut-offs for age and sex [30]. Pilomotor nerve fibre density was reduced in 95% of the MSA group and 78% of the PD group using pan-neuronal marker PGP, 100% of MSA and 97% of PD using the cholinergic marker VIP, and 86% of MSA and 69% of PD using adrenergic marker DβH [28]. Sudomotor innervation was reduced in 72% of patients with MSA and 50% of patients with PD with PGP, and 91% of patients with MSA and 60% of patients with PD with VIP [7].

**Fig. 3:**
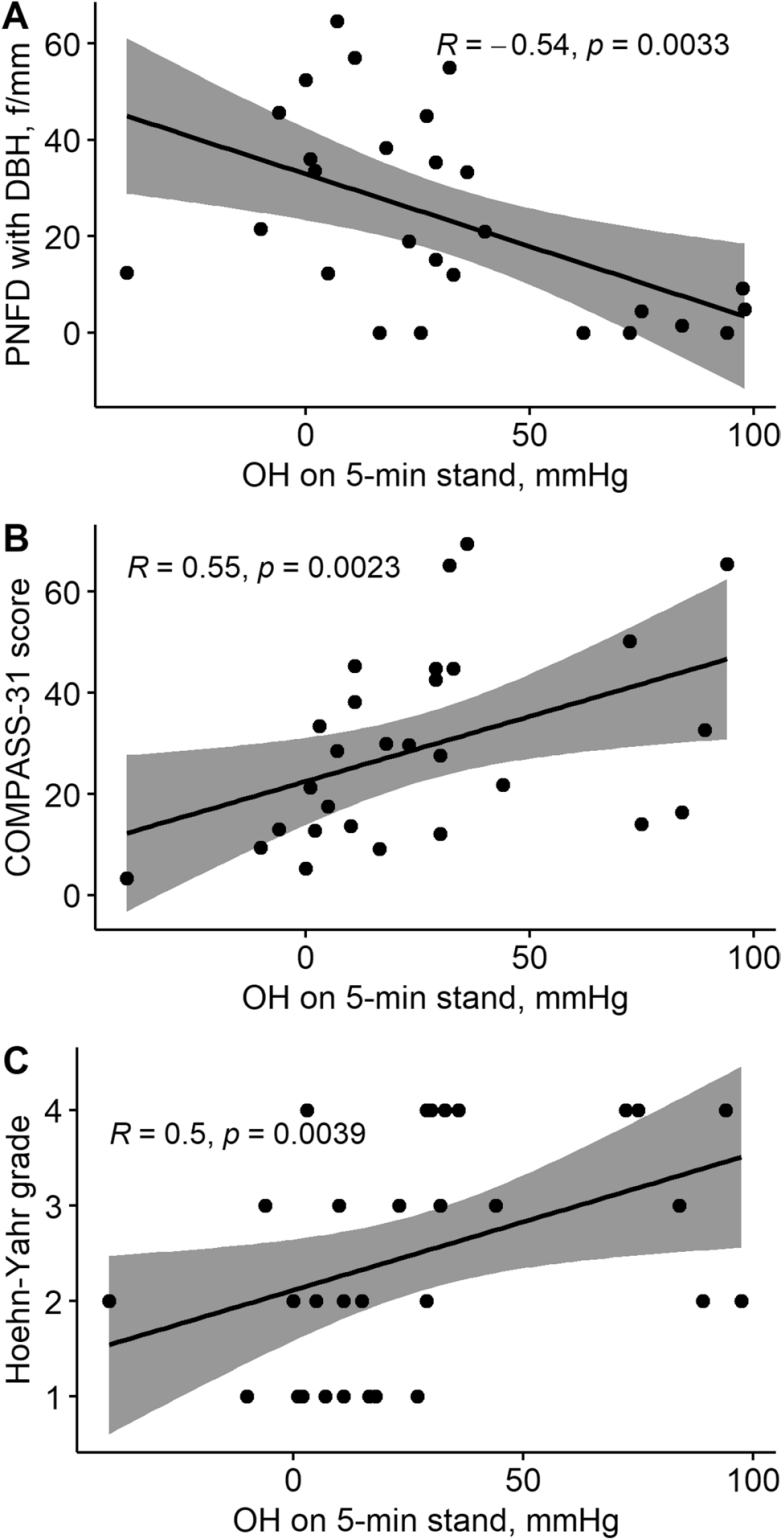
Severity of OH correlated with cutaneous adrenergic denervation, patient reported symptoms and disease severity. Severity of OH, as measured by the fall in systolic blood pressure on standing, correlated with pilomotor nerve fibre density with the adrenergic marker DβH (**A**). OH severity also correlated with patient reported autonomic symptoms on the COMPASS-31 questionnaire (**B**) and disease status as measured by the Hoehn–Yahr scale (**C**)

IENFD was reduced in nOH and no-OH groups compared with controls (*P* < 0.001). Pilomotor innervation was reduced in the nOH group compared with controls with PGP, VIP and DβH (*P* < 0.001) and was also reduced in the no-OH groups versus controls using PGP (*P* = 0.01) and VIP (*P* = 0.002) (Table 4). Sudomotor innervation with PGP was lower in nOH groups compared with controls (*P* < 0.001). Sudomotor innervation with VIP was lower in the nOH (0.9 [0.1–1.3] fibres/mm) compared with the no-OH (1.6 [1.4–1.8] fibres/mm, *P* = 0.01) and control groups (2.1 [1.9–2.7] fibres/mm, *P* = 0.001) (Fig. 3).

### Cardiac MIBG

**Fig. 4:**
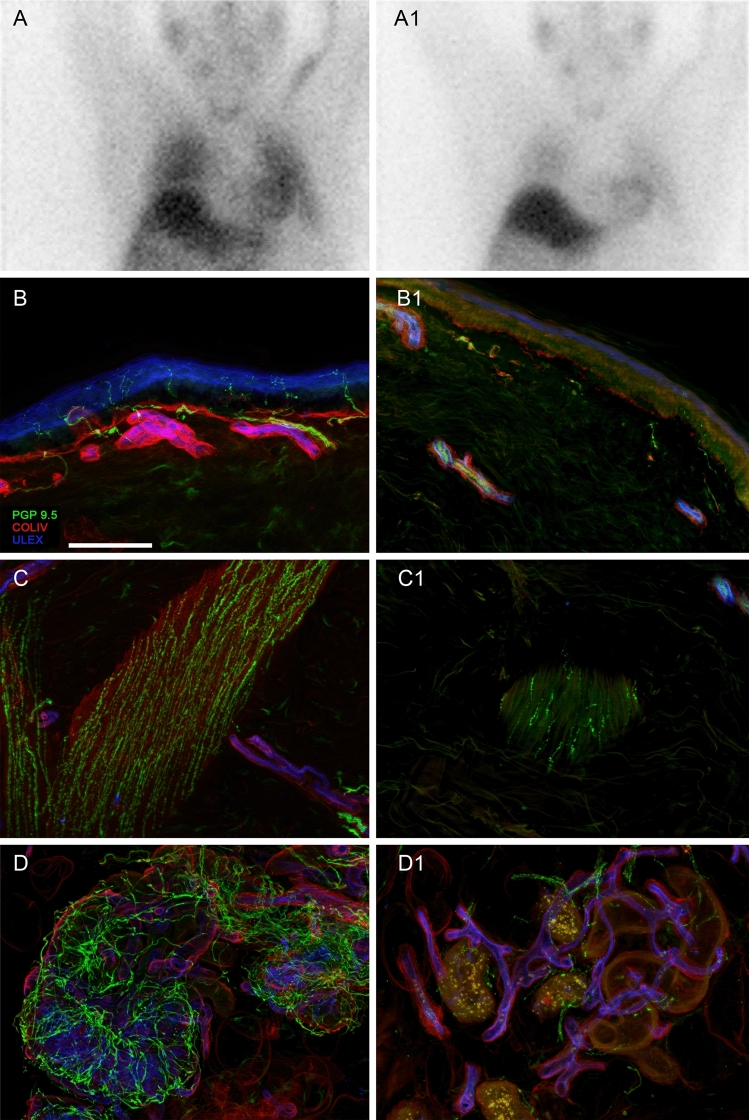
Cardiac MIBG and cutaneous innervation in a patient with MSA (case 1). MIBG study performed as part of clinical work-up. Images acquired 10 min (**A**) and 4 h (**A1**) post-injection showed good tracer uptake in the myocardium with a heart to mediastinum ratio of 1.73, indicating preserved postganglionic innervation. However, punch skin biopsies from the distal legs bilaterally revealed severe cutaneous denervation with marked loss of intraepidermal (0.1–1.1 fibres/mm), pilomotor (15.2–32.0 fibres/mm) and sudomotor fibres (0.4 nm/µm^3^) with the pan-neuronal marker PGP (**B**–**D**, control; **B1**–**D1**, patient), and a complete loss of pilomotor and sudomotor fibres with the cholinergic marker VIP (data not shown). Scale bar: 100 µm

MIBG was performed as part of the clinical work-up in two patients: one with PD and one with MSA. The patient with PD was a 56-year-old man recruited less than 2 years after disease onset, with no symptoms of orthostatic tolerance and normal supine and standing blood pressure. He had a normal heart: mediastinum ratio on MIBG (1.4), with normal mean IENFD (10.5 fibres/mm, normal 95% cut-off for age and sex > 8.8 fibres/mm), normal sudomotor innervation with PGP (5.1 nm/μm^3^, normal > 3.0 nm/μm^3^) and VIP (2.4 nm/μm^3^, normal > 1.7 nm/μm^3^) and relatively preserved pilomotor density, just below normal cut-off values of PGP (65.1 fibres/mm, normal > 67.2 fibres/mm), VIP (39.6 fibres/mm, normal > 44.4 fibres/mm) and DBH (36.6 fibres/mm, normal 38.7 fibres/mm). The patient with MSA was a 66-year-old man recruited 6 years after disease onset, with cardiovascular autonomic failure and neurogenic OH. Mean supine blood pressure was 125/81 mmHg falling to 104/54 mmHg at 3 min, and 95/57 mmHg at 5 min, with an abnormal BP profile with the Valsalva manoeuvre (absent phase II-late and phase IV overshoot, prolonged PRT of 15.3 s). He had a normal supine plasma noradrenaline level (224 pg/mL) and normal heart: mediastinum ratio of 1.7 on cardiac MIBG (Fig. 4). However, we found severe postganglionic sudomotor dysfunction on DST and cutaneous somatic and autonomic denervation using PGP (IENFD 0.6 fibres/mm, normal 95% cut off for age and sex > 8.2 fibres/mm), pilomotor nerve fibre density (23.6 fibres/mm, normal > 67.2 fibres/mm) and sudomotor innervation (0.4 nm/µm^3^, normal > 3.0 nm/μm^3^). There was a complete loss of pilomotor and sudomotor fibres with the cholinergic marker VIP. Our study findings and these cases highlight how peripheral autonomic denervation does not occur uniformly across disease groups and individual patients.

### Correlations between OH, cutaneous autonomic denervation, patient reported outcomes, disease severity and disease duration

There was a strong correlation between the severity of nOH, as measured by the fall in systolic blood pressure at 5 min standing, and pilomotor adrenergic innervation (*ρ* = −0.54, *P* = 0.003). The fall in systolic blood pressure on standing also correlated significantly with patient reported autonomic symptoms on the COMPASS-31 (*ρ* = 0.55, *P* = 0.002), and disease severity as measured by the Hoehn–Yahr scale (*ρ* = 0.50, *P* = 0.004) (Fig. 3). Disease duration correlated with the fall in systolic blood pressure on standing (*ρ* = 0.59, *P* < 0.001), intraepidermal innervation (*ρ* = −0.41, *P* = 0.002), pilomotor adrenergic innervation, *ρ* = −0.41, *P* = 0.006), sudomotor cholinergic innervation, *ρ* = −0.51, *P* < 0.001) and Hoehn–Yahr grade (*ρ* = −0.46, *P* = 0.001). Supine noradrenaline did not correlate with any quantitative cardiovascular or sudomotor testing parameters or cutaneous innervation.

## Discussion

Our study has revealed insights into the pathophysiology of nOH and cardiovascular autonomic failure in MSA and PD with clinically relevant implications. We found that in most patients with MSA and PD, nOH rarely occurs in isolation, but is typically part of a broader more generalised impairment of cardiovascular autonomic reflexes, reflecting more widespread cardiovascular autonomic failure. Consistent with previous studies, we confirmed that nOH contributes significantly to functional disability in both diseases [5]. Importantly, we observed that the severity of nOH correlated strongly with cutaneous adrenergic denervation, patient reported autonomic symptoms and Hoehn–Yahr grade. To our knowledge, this is the first study demonstrating an association between cutaneous adrenergic denervation and the presence and severity of nOH in PD and MSA. Although cutaneous adrenergic fibres may play a limited direct role in systemic blood pressure regulation, their loss could be a marker for more widespread peripheral adrenergic denervation, which may critically impair orthostatic blood pressure control.

Our findings suggest that both PD and MSA exhibit evidence of postganglionic autonomic involvement. This appears to contrast with the traditional view that autonomic failure in MSA is predominantly preganglionic, while PD is characterised by more postganglionic pathology. However, emerging evidence indicates that the distinction may not be absolute. Several studies have reported postganglionic involvement in MSA, suggesting a spectrum rather than a strict dichotomy of autonomic involvement [6, 8, 9, 31]. In our cohort, the degree of postganglionic autonomic denervation correlated with the severity of cardiovascular autonomic failure, irrespective of clinical diagnosis, supporting the hypothesis that postganglionic denervation contributes to pathophysiology of nOH in both diseases. This observation may have important therapeutic implications, particularly regarding treatments aimed at enhancing noradrenaline availability. We acknowledge that clearer separation of groups, particularly stratifying by disease duration or using additional markers of central versus peripheral involvement, may help refine this interpretation. We did not perform further subgroup analyses based on disease duration owing to the small sample size of individual groups. However, our study cohort included four patients with MSA with < 1 year disease duration. All had reduced pilomotor innervation with PGP, VIP and DβH, and 50% had reduced sudomotor innervation with either PGP or VIP, providing evidence for postganglionic autonomic denervation even in early MSA. We believe our study adds important evidence to the ongoing debate on the pathophysiology of cardiovascular autonomic failure and nOH in MSA, with potential implications for treatment. Our results partially contrast with the study from Donadio et al., which reported predominantly somatic fibre involvement with preserved autonomic innervation in MSA-P [32]. However, closer examination of their supplementary data reveals sudomotor innervation at the distal leg was reduced in both patients with MSA-P and patients with PD and nOH, supporting the notion that postganglionic autonomic involvement in MSA may be more common than previously appreciated.

We found supine plasma noradrenaline levels were significantly higher in MSA versus PD with nOH subgroups, in keeping with previous studies [33, 34], and in fact, supine noradrenaline was the only biomarker to differ significantly amongst the MSA and PD with nOH subgroups. However, plasma noradrenaline levels did not correlate with cutaneous adrenergic innervation, underscoring the complexity of using plasma catecholamines as biomarkers of sympathetic integrity. The relationship between plasma noradrenaline levels and sympathetic innervation and activity is complex, and resting plasma supine noradrenaline levels are likely to reflect both production from postganglionic pre-synaptic terminals, cellular reuptake, metabolism and storage. Peripheral adrenergic denervation is likely to contribute to abnormal responses to physiological and pharmacological stimuli, including novel therapeutic agents for nOH that aim to enhance availability of noradrenaline [35–39]. Previous studies using surrogate markers of postganglionic autonomic denervation to try predict responses to novel agents for nOH have generated conflicting results. Palma et al.’s study of 20 patients with nOH found supine noradrenaline levels predicted pressor response to droxidopa, a pro-drug of noradrenaline, whereas Shibao et al.’s larger study of 99 patients with nOH found neither plasma noradrenaline nor cardiac innervation on MIBG were good predictors for response to the noradrenaline reuptake inhibitor atomoxetine [36, 38]. While plasma noradrenaline levels can provide some information regarding sympathetic function, they do not provide a direct measure of postganglionic denervation, highlighting the need for more specific biomarkers.

Post-mortem studies have shown a loss of sympathetic preganglionic cells in the intermediolateral cell column of the thoracolumbar spinal cord in patients with PD and MSA with autonomic failure compared with patients without autonomic failure and normal controls [40], but a loss of postganglionic cardiac adrenergic fibres in patients with PD only [41]. In vivo cardiac scintigraphy studies have also demonstrated cardiac uptake of noradrenaline analogues tends to be spared in MSA compared with PD [4, 42, 43], although these studies do not always reliably distinguish between individuals with MSA and PD [5, 44]. The differences in noradrenergic innervation at other bodily sites as assessed by ^18^F-dopamine positron emission topographic scans and post-mortem neurochemical data suggests that the difference between patients with Lewy body synucleinopathies, including patients with PD, and MSA is cardioselective, and there were no differences between norepinephrine levels in the sympathetic ganglia and a number of other organs studied [45]. In our study, cardiovascular autonomic testing did not differ significantly between MSA with nOH and PD with nOH subgroups, in agreement with previous reports [5, 46], suggesting that cardiovascular autonomic testing alone cannot reliably distinguish between these two diseases when OH is present. However, some trends were noted: patients with MSA and nOH showed more severe orthostatic hypotension and diminished blood pressure response to isometric exercise, whereas the patients with PD and nOH exhibited lower Valsalva ratios and reduced heart rate responses to isometric exercise, suggesting relatively greater cardiac adrenergic denervation.

In this exploratory multicentre study, we chose to study 3-mm skin distal leg biopsies, for which there is excellent safety data previously reported, with no serious side effects reported in over 35,000 biopsies performed over 15 years across ten laboratories [27]. We chose to quantify cutaneous sudomotor and pilomotor autonomic innervation as our group and others have previously established and validated methods to quantify innervation to these autonomic adnexa [27, 28, 47]. The variability and complexity of cutaneous vasculature and its innervation has limited previous attempts to quantify vascular innervation. Sohn et al. recently outlined a method of quantifying cutaneous vessel innervation in 20 patients with diabetes and 19 controls, and had similar findings to our study, showing individuals with nOH had lower vascular innervation than those without nOH, with significant correlations between neurovascular density and cardiovascular autonomic biomarkers, including blood pressure drop during head-up tilt, blood pressure overshoot on phase IV of the Valsalva manoeuvre and autonomic symptom scores [48].

Strengths of our study include the comprehensive and systematic assessment of patients with cardiovascular, sudomotor, plasma noradrenaline and in-depth analysis of autonomic innervation on skin biopsy, with comparisons to normative data, to explore the relationship between postganglionic autonomic denervation and autonomic failure. Whilst the numbers studied are not large, each subject was comprehensively evaluated with a battery of well-defined quantitative autonomic and cutaneous biomarkers. We believe the study is of interest as it provides insight to the relationship between postganglionic autonomic denervation and cardiovascular autonomic failure using non-invasive and minimally invasive tests performed in vivo. One of the limitations of our study is that none of the patients underwent a post-mortem to confirm the clinical diagnosis, and there is overlap between the clinical criteria of MSA and PD with nOH. Nevertheless, all patients were assessed by neurologists with movement disorder and autonomic expertise, and were followed-up for 2 years from initial recruitment, with exclusion of subjects who had an alternative clinical diagnosis on follow-up.

In our cohort, the degree of postganglionic denervation correlated with the severity of cardiovascular autonomic failure, irrespective of clinical diagnosis, supporting the notion that postganglionic pathology contributes to neurogenic orthostatic hypotension (nOH) in both diseases.

In summary, our findings suggest that postganglionic adrenergic denervation contributes to cardiovascular autonomic failure in both PD and MSA. The association between cutaneous adrenergic fibre loss, the severity of nOH and patient-reported outcomes highlights the potential clinical relevance of peripheral adrenergic denervation. These insights may influence therapeutic strategies for nOH and support the development of more targeted treatments in these patient populations.

## Supplementary Information

Below is the link to the electronic supplementary material.

Supplementary file1 (DOCX 21 KB)

## Data Availability

The data that support the findings of this study are available on request.
