# Supplementary material for: Cardiovascular autonomic failure correlates with cutaneous autonomic denervation in PD and MSA
Source: Clin Auton Res. 2025 Sep 12;36(1):65–77. doi: 10.1007/s10286-025-01154-4 (PMC12982203; doi:10.1007/s10286-025-01154-4)
Supplement: Supplementary file 1 — Supplementary file1 (DOCX 21 KB) [file 10286_2025_1154_MOESM1_ESM.docx]

**Supplementary Table 1. Clinical and cardiovascular testing in patients with and without OH**

|  | Median (IQR) | | *P*-Value |
| --- | --- | --- | --- |
|  | OH (n=23) | No OH (n=20) |  |
| Clinical details |  |  |  |
| Age, y | 64 (56-71) | 63 (60-69) | .90 |
| Sex (F/M) | 5/18 | 9/11 | .20 |
| Disease duration, months | 33 (20-44) | 21 (11-23) | .002 |
| Hoehn-Yahr | 3.5 (2.75-4) | 1 (1-2) | <.001 |
| COMPASS-31 | 33 (25-48) | 17 (9-30) | .006 |
| SFN-SIQ | 10 (7-11) | 7 (5-10) | .16 |
|  |  |  |  |
| Cardiovascular testing |  |  |  |
| Δ SBP on standing |  |  |  |
| 1 min, mmHg | 37 (19-56) | 1 (-4-16) | <.001 |
| 3 min, mmHg | 37 (21-70) | 4 (-2 to 9) | <.001 |
| 5 min, mmHg | 35 (29-74) | 4 (-1 to 11) | <.001 |
| Isometric exercise |  |  |  |
| Δ SBP, mmHg | 5 (-2^a^ to 15) | 22 (14-26) | <.001 |
| Δ HR, bpm | 4 (1-6) | 11 (7-14) | .004 |
| HR_DB_, bpm | 7 (4-11) | 12 (8-18) | .048 |
| Valsalva ratio | 1.10 (1.03-1.18) | 1.33 (1.21-1.42) | .02 |
| Pressure recovery time, s | 14.5 (8.9-22.4) | 2.7 (2.5-2.8) | .02 |
|  |  |  |  |
| Supine noradrenaline, pg/ml | 242 (228-293) | 232 (224-239) | .72 |
| Δ noradrenaline on tilt, pg/ml | 11 (6-18) | 93 (80-106) | .06 |

SBP, systolic blood pressure; HR, heart rate
